# Basal mitophagy is widespread in *Drosophila* but minimally affected by loss of Pink1 or parkin

**DOI:** 10.1083/jcb.201801044

**Published:** 2018-05-07

**Authors:** Juliette J. Lee, Alvaro Sanchez-Martinez, Aitor Martinez Zarate, Cristiane Benincá, Ugo Mayor, Michael J. Clague, Alexander J. Whitworth

**Affiliations:** 1 Medical Research Council Mitochondrial Biology Unit, University of Cambridge, Cambridge, England, UK; 2 Department of Biochemistry and Molecular Biology, University of the Basque Country, Leioa-Bizkaia, Spain; 3 Department of Molecular and Cellular Physiology, Institute of Translational Medicine, University of Liverpool, Liverpool, England, UK

## Abstract

The Parkinson’s disease factors PINK1 and parkin are strongly implicated in stress-induced mitophagy in vitro, but little is known about their impact on basal mitophagy in vivo. We generated transgenic *Drosophila melanogaster* expressing fluorescent mitophagy reporters to evaluate the impact of *Pink1/parkin* mutations on basal mitophagy under physiological conditions. We find that mitophagy is readily detectable and abundant in many tissues, including Parkinson’s disease–relevant dopaminergic neurons. However, we did not detect mitolysosomes in flight muscle. Surprisingly, in *Pink1* or *parkin* null flies, we did not observe any substantial impact on basal mitophagy. Because these flies exhibit locomotor defects and dopaminergic neuron loss, our findings raise questions about current assumptions of the pathogenic mechanism associated with the PINK1/parkin pathway. Our findings provide evidence that Pink1 and parkin are not essential for bulk basal mitophagy in *Drosophila*. They also emphasize that mechanisms underpinning basal mitophagy remain largely obscure.

## Abstract (precis)

PINK1/parkin are key mediators of stress-induced mitophagy in vitro, but their impact on basal mitophagy in vivo is unclear. Novel *Drosophila* reporter lines reveal abundant mitophagy in many tissues, including dopaminergic neurons, that is unaffected by loss of PINK1/parkin.

## Introduction

Mitochondria are essential organelles that perform many critical metabolic functions but are also a major source of damaging reactive oxygen species and harbor proapoptotic factors. Multiple homeostatic processes operate to maintain mitochondrial integrity; however, terminally damaged organelles are degraded through the process of targeted mitochondrial autophagy (mitophagy) to prevent potentially catastrophic consequences. Such homeostatic mechanisms are particularly important for postmitotic, energetically demanding tissues such as nerves and muscles. Two proteins linked to Parkinson’s disease (PD), parkin, a cytosolic ubiquitin E3 ligase, and PINK1, a mitochondrially targeted kinase, have been shown to play key roles in mitophagy.

PINK1/Parkin-mediated mitophagy has been intensively investigated, and many of the molecular mechanisms have been elucidated (Pickrell and Youle, 2015; Yamano et al., 2016). In brief, upon loss of mitochondrial membrane potential as occurs in damaged or dysfunctional mitochondria, PINK1 accumulates on the outer mitochondrial membrane (OMM) and initiates the mitophagy signal by phosphorylating both ubiquitin and parkin. This promotes parkin’s E3-ligase activity, thereby depositing more ubiquitin for subsequent phosphorylation. This process acts as a feed-forward mechanism ultimately decorating the OMM with phosphoubiquitin chains that are then recognized by ubiquitin adaptor proteins leading to the engulfment of the depolarized mitochondria by autophagosomes.

As a pathogenic mechanism, failure in mitophagy offers an attractive explanation for multiple, longstanding observations that implicate mitochondrial defects in the pathogenicity of PD, such as systemic mitochondrial complex I deficits and high levels of mitochondrial DNA mutations in PD patients (Schapira et al., 1989; Bender et al., 2006; Kraytsberg et al., 2006; Exner et al., 2012; Dias et al., 2013). In addition, the unique physiological characteristics of *substantia nigra* neurons, such as their extensive arborization, myriad synaptic connections, and continuous pacemaking activity, place an extreme demand on mitochondrial function to meet their high energy and calcium buffering requirements. This may explain the selective vulnerability of these neurons to loss of mitochondrial integrity (Sulzer and Surmeier, 2013).

Many of the molecular details of PINK1/parkin-induced mitophagy have been elaborated in cultured cells that overexpress parkin coupled with acute mitochondrial depolarization or toxification (Pickrell and Youle, 2015; Yamano et al., 2016). However, relatively little is known about mitophagy under physiological conditions in vivo (Cummins and Gotz, 2017; Rodger et al., 2017; Whitworth and Pallanck, 2017). One of the limitations to studying mitophagy in vivo has been the paucity of suitable reporters. Recently, two in vivo mitophagy reporter models have been described in mice (Sun et al., 2015; McWilliams et al., 2016). One uses a mitochondrial matrix–targeted pH-sensitive variant of GFP (mt-Keima), whereas the other uses a tandem GFP-mCherry fusion protein targeted to the OMM (called mito-QC). Both systems exploit pH-sensitive properties of mKeima and GFP, respectively, to enable the differential labeling of mitochondria in the acidic microenvironment of the lysosome as a proxy endpoint readout. Initial studies on these two reporter lines have revealed a surprisingly widespread and heterogeneous distribution of basal mitophagy, but the involvement of PINK1 and parkin has not been addressed.

We have generated *Drosophila melanogaster* lines expressing these mitophagy reporters to provide the first global view of the prevalence of mitophagy across the organism and to determine the relative contribution of Pink1 and parkin to basal mitophagy. Analyzing mitophagy in *Drosophila* offers a valuable opportunity to interrogate the physiological requirement of Pink1/parkin in this process in vivo. In contrast to most mammalian models, loss of *Drosophila Pink1* or *parkin* leads to robust phenotypes in locomotor activity and dopaminergic (DA) neuron loss (Greene et al., 2003; Clark et al., 2006; Park et al., 2006), indicating their critical function in the related neuromuscular tissues. We find that although basal mitophagy is widespread in many tissues in *Drosophila*, the incidence of mitolysosomes, and hence, basal mitophagy, is unaffected by loss of Pink1 or parkin. Moreover, flight muscle, which shows the most robust disruption upon *Pink1/parkin* mutation (Greene et al., 2003, 2005; Clark et al., 2006; Park et al., 2006), does not reveal a detectable mitophagy signal. Hence, we propose that Pink1 and parkin are largely dispensable for basal mitophagy and have mitophagy-independent functions in neuromuscular tissues in *Drosophila*.

## Results and discussion

### Validation of mitophagy reporters in *Drosophila*

We first generated GAL4/UAS-inducible transgenic lines to express either mito-QC or mt-Keima. Multiple lines were established and immunoblotting confirmed comparable expression between lines (Fig. S1, A and B). Expression of these reporters in all lines appeared benign and did not noticeably affect development or viability. To ensure that the expression of these reporters did not overtly interfere with normal cellular functions in vivo, particularly in the sensitive neuromuscular systems of interest here, we analyzed locomotor activity in flies expressing high levels of the mito-QC or mt-Keima in all tissues and observed no significant impact (Fig. S1, C–F).

**Figure 1.**
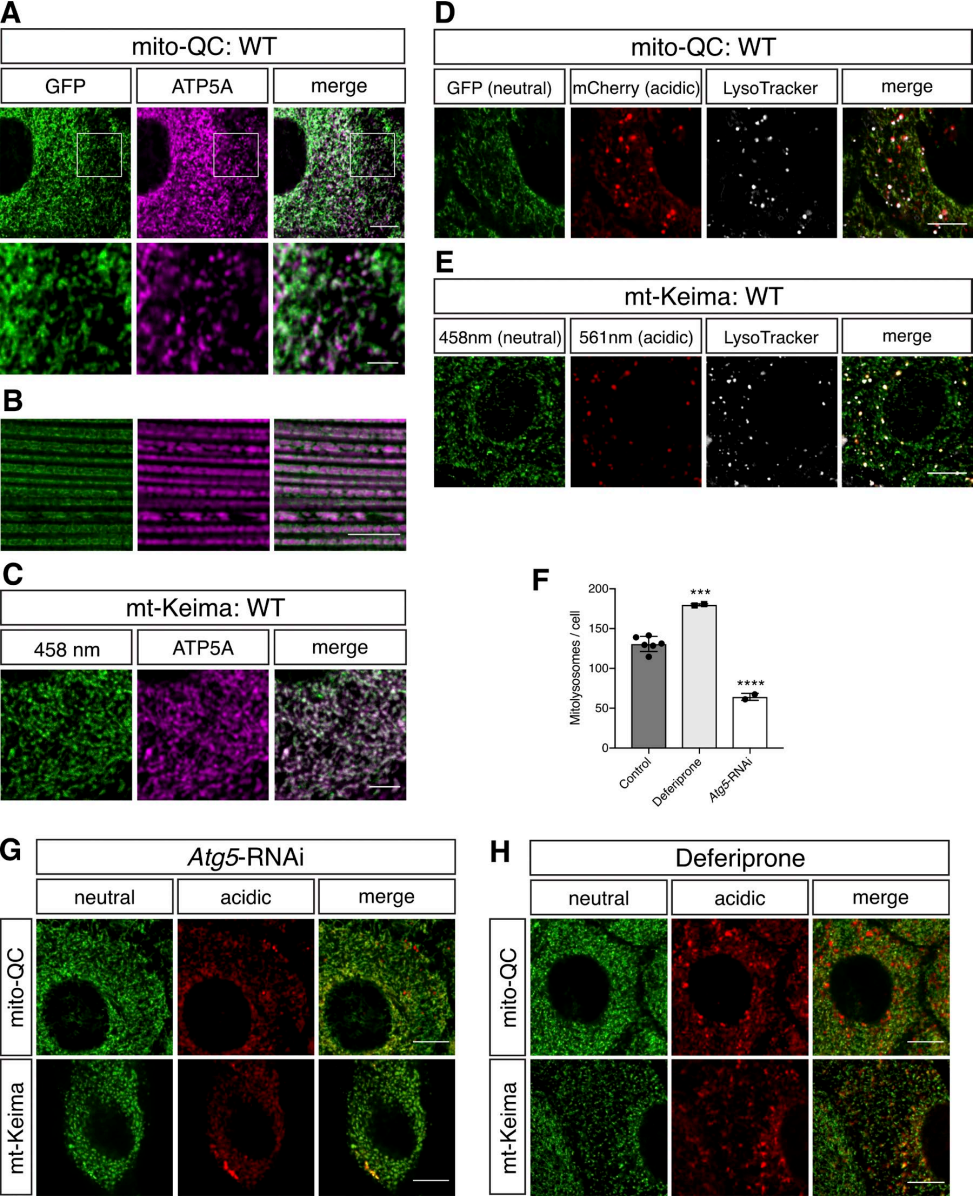
**Validation of mito-QC and mt-Keima mitophagy reporters. (A–C)** Immunohistochemical and confocal imaging analysis of larval epidermal cells (A and C) and adult flight muscle (B). (A and B) Native GFP fluorescence (green) and (C) 458-nm-stimulated fluorescence (neutral pH) were compared with a mitochondrial protein distribution by immunostaining with anti-ATP5A (magenta). **(D and E)** Confocal live imaging analysis of larval epidermal cells for mito-QC (D) and mt-Keima (E) neutral and acidic pH spectra costained for LysoTracker to mark lysosomes. Fluorescence corresponding to neutral pH and acidic pH are shown in green and red, respectively. LysoTracker is shown in white. **(F)** Quantification of mitolysosomes in mito-QC larval epidermal cells shown in G and H. Chart shows mean ± SD of *n* = 6 animals for (WT) control, *n* = 2 animals for deferiprone and *Atg5*-RNAi expression. Number of cells analyzed: epidermis, WT = 29 cells, deferiprone and *Atg5*-RNAi = 14 cells each. Statistical significance determined by one-way ANOVA with Sidak’s post-hoc test; ***, P < 0.001; ****, P < 0.0001. **(G and H)** Confocal imaging analysis of larval epidermal cells for mito-QC and mt-Keima upon expression of an *Atg5*-RNAi transgene to inhibit autophagy (G) or exposure to the iron chelator deferiprone (65 µM) to induce mitophagy (H). Bars: (A, top; and B, D, E, G, and H) 10 µm; (A, bottom; and C) 4 µm. Genotypes analyzed were *da-GAL4/UAS-mito-QC* (A, D, and H), *Mef2-GAL4/UAS-mito-QC* (B), *tub-GAL4*, *UAS-mt-Keima* (C, E, and H), and *da-GAL4, UAS-mito-QC/UAS*-*Atg5-*RNAi and *tub-GAL4*, *UAS-mt-Keima*/*UAS*-*Atg5-*RNAi (G).

Previous work has demonstrated the appropriate targeting of mito-QC and mt-Keima to mitochondria in vitro and in vivo, and that “spectral shifted” puncta of more acidic conditions indeed colocalized with lysosomes (Katayama et al., 2011; Allen et al., 2013). Nevertheless, we also sought to verify these conditions in our *Drosophila* lines. As expected, we observed substantial colocalization of mito-QC and mt-Keima with the mitochondrial protein ATP5A in two tissues highly amenable for mitochondrial imaging analysis, larval epidermal cells and adult flight muscle (Fig. 1, A–C). Interestingly, the outer membrane targeted mito-QC shows a nonuniform distribution across the network in epidermal cells, which reflects the dynamic and heterogeneous nature of the mitochondrial network. In contrast, the matrix localized mt-Keima more precisely colocalizes with ATP5A immunostaining.

The spectral shift that marks mitolysosomes of mito-QC occurs by the selective quenching of GFP, but not mCherry, resulting in “red-only” puncta (Fig. 1 D). Similarly, for mt-Keima, the fluorescence spectrum shifts to reflect an increased signal from excitation at 561 nm when under more acidic conditions (Fig. 1 E). With both mitophagy reporters, we observed a striking colocalization of “mitophagy” puncta with LysoTracker (Fig. 1, D and E).

To further validate these puncta as mitolysosomes, we assessed their formation in tissue lacking the canonical autophagy pathway generated by knockdown of the key autophagy factor *Atg5*. Mitolysosome signal reported by both mito-QC and mt-Keima were significantly reduced in *Atg5*-RNAi epidermal cells (Fig. 1, F and G), consistent with their appearance reflecting mitophagy. Moreover, we sought to demonstrate that these reporters were sensitive enough to reveal induced mitophagy. Although sustained treatment with mitochondrial depolarizing agents, such as carbonyl cyanide 3-chlorophenylhydrazone, was not feasible in vivo, it was previously reported that iron chelation by deferiprone induced mitophagy reported by mito-QC (Allen et al., 2013). Feeding animals deferiprone, we observed an increase in mitolysosomes reported by mito-QC and mt-Keima (Fig. 1, F and H). Collectively, these findings validate the mitophagy signal of mito-QC and mt-Keima as faithful reporters of mitolysosomes in *Drosophila*, similarly to previously characterized mouse models (Sun et al., 2015; McWilliams et al., 2016).

### Mitophagy is widespread in multiple *Drosophila* tissues

**Figure 2.**
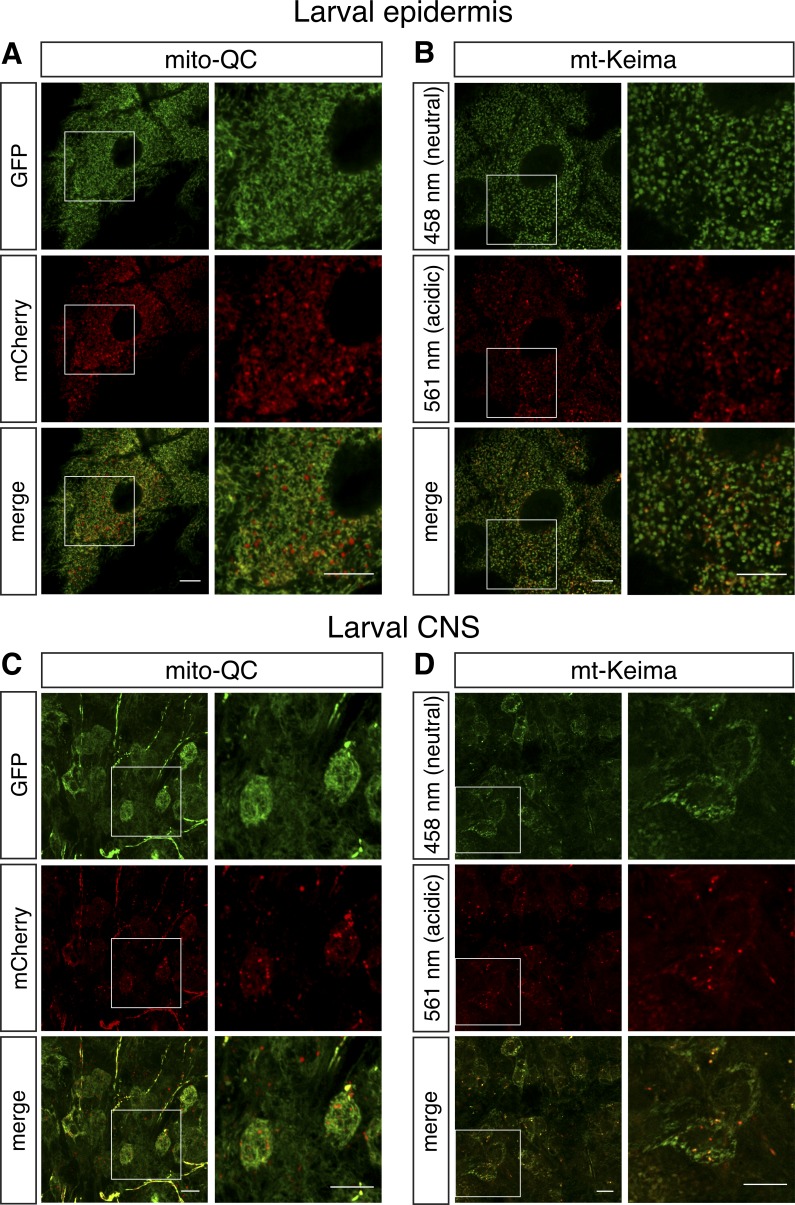
**Comparison of mito-QC and mt-Keima mitophagy reporters in larval tissues. (A–D)** Confocal microscopy analysis of larval epidermal cells (A and B) and the ventral ganglion of the CNS (C and D), visualizing mito-QC (fixed) and mt-Keima (live) as indicated. Fluorescence corresponding to neutral pH and acidic pH are shown in green and red, respectively. Genotypes analyzed were *da-GAL4/UAS-mito-QC* (A and C) and *tub-GAL4*, *UAS-mt-Keima* (B and D). Bars, 10 µm.

We next determined the prevalence of mitolysosomes under basal conditions in multiple tissues. In larvae, we analyzed epidermal cells, because they have an elaborate mitochondrial morphology, and the ventral ganglion, a major portion of the central nervous system (CNS). Here, we directly compared the mitolysosome signal reported by mito-QC and mt-Keima. Microscopic analysis revealed that mitolysosomes are abundant under basal conditions in both epidermal cells and CNS (Fig. 2, A–D). In contrast, mitolysosomes were almost undetectable in larval body wall muscles (unpublished data). Importantly, mito-QC and mt-Keima revealed a very similar abundance and distribution of mitolysosomes, substantiating their utility as equivalent mitophagy reporters.

During this stage of analysis, we found that the mt-Keima signal, which was already markedly weaker than that generated by mito-QC, was rapidly bleached upon extended exposure or repeated scanning. Repeated imaging was necessary to achieve adequate penetration into complex tissue and for z-stack (3D) reconstruction for mitolysosome quantification. For this reason, the subsequent analyses mainly focused on analyzing mito-QC.

**Figure 3.**
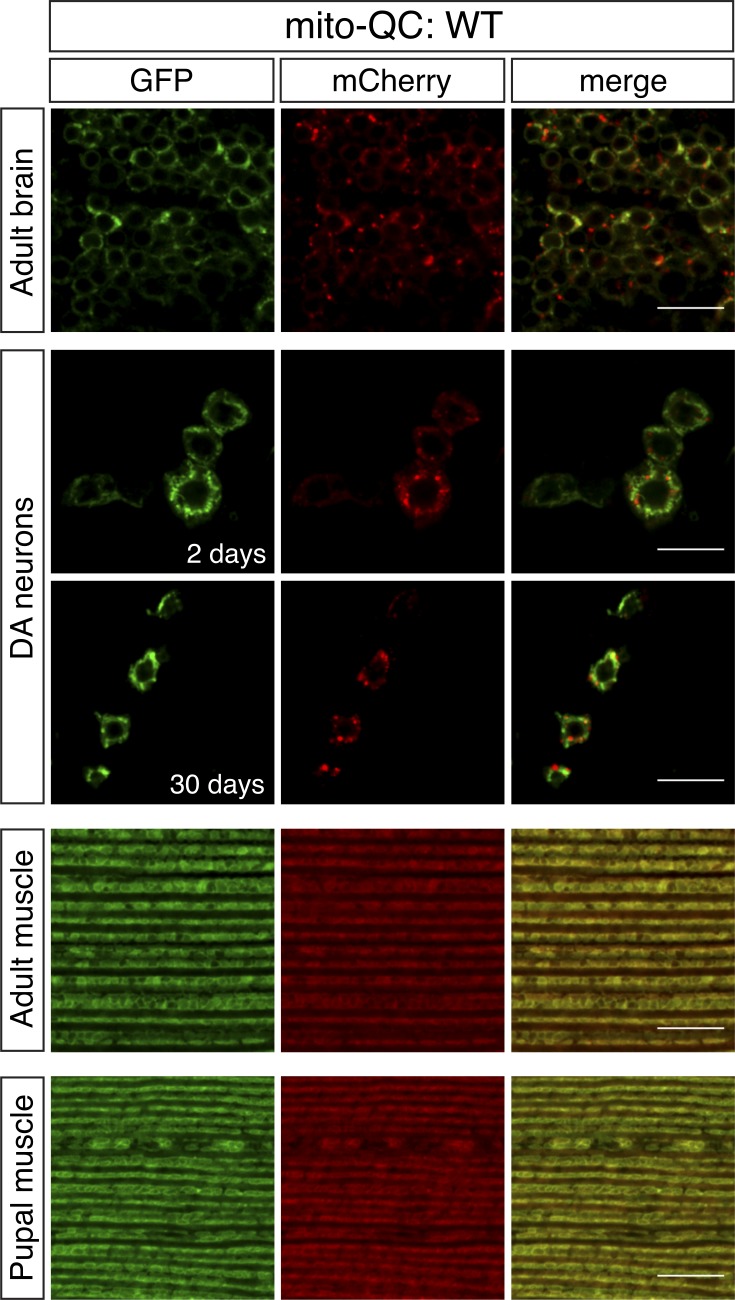
**Mitolysosome analysis with mito-QC in adult tissues.** Confocal microscopy analysis of mito-QC reporter in WT adult brain (2 d old), DA neurons at 2 and 30 d old, flight muscle (2 d old), and preadult (pupal) flight muscle, as indicated. Mitolysosomes are evident as GFP-negative/mCherry-positive (red-only) puncta. Quantification of mitolysosomes is shown in Figs. 4 and 5. Genotypes analyzed were *UAS-mito-QC/+;nSyb-GAL4/*+ (adult brain), *UAS-mito-QC/+;TH-GAL4/*+ (DA neurons), and *Mef2-GAL4/UAS-mito-QC* (adult and pupal muscle). Bars, 10 µm.

We next analyzed adult tissues where postmitotic, highly energetic tissues would be expected to accumulate more mitochondrial damage and therefore likely undergo more mitophagy. Again, we observed abundant mitolysosomes, widespread in a medial region of the posterior protocerebrum (adult brain; Fig. 3). PD is associated with the preferential degeneration of DA neurons, which is reproduced in multiple *Drosophila* models of PD, including by cell-autonomous loss of *Pink1/parkin* (Hewitt and Whitworth, 2017). Mito-QC was selectively expressed in DA neurons via the tyrosine hydroxylase (TH) GAL4 driver. Analyzing the PPL1 cluster, which has most consistently been shown to be affected in *Pink1/parkin* mutants, we observed a substantial mitophagy signal in DA neurons (Fig. 3). The amount of mitophagy signal did not markedly increase with ageing.

Somewhat surprisingly, but consistent with observations in larvae, we did not observe any appreciable mitolysosomes in 2-d-old flight muscle (Fig. 3). This is notable for two reasons. First, adult flight muscle in particular has extremely abundant mitochondria necessary to power flight. Second, *Pink1* and *parkin* mutant *Drosophila* display robust degeneration of flight muscles. It could be reasonably assumed that this degeneration results from a failure in mitophagy induction. The lack of mitophagy signal is especially surprising given the abundant mitochondria and apparent abundant lysosomes in this tissue (Demontis and Perrimon, 2010).

We reasoned that analyzing adult tissue could possibly have missed a critical period when mitophagy is active and required for muscle integrity, such as when the tissue is being formed in development. To address this, we analyzed flight muscle of the pupal stage when disruption of muscle integrity first occurs in *Pink1/parkin* mutants (Greene et al., 2005; Clark et al., 2006). However, we also could not detect mitolysosomes at these earlier time points (Fig. 3). Collectively, from these observations, we surmise that mitophagy is abundant in various tissues during development and in adult flies, especially in neuronal tissue, but is not detectable in muscle tissue at either stage.

### Pink1 and parkin are largely dispensable for basal mitophagy in *Drosophila*

**Figure 4.**
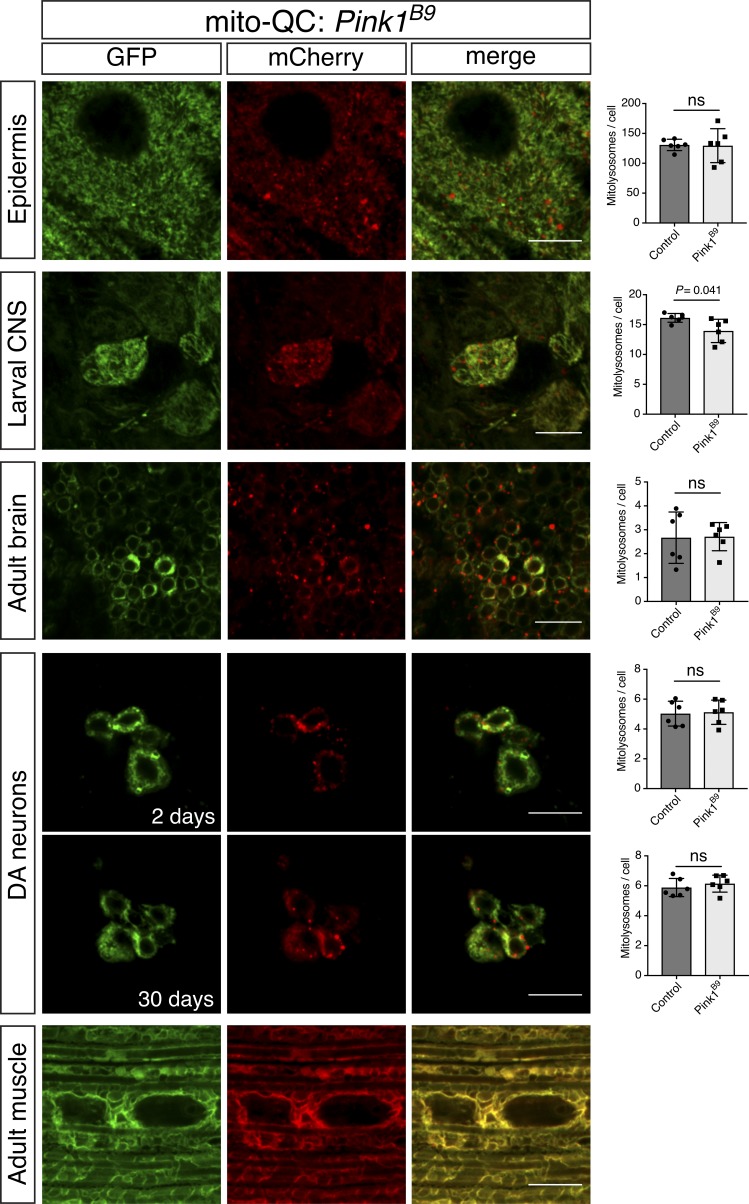
**Basal mitophagy is minimally affected in *Pink1* mutants.** Confocal microscopy analysis of mito-QC reporter in *Pink1^B9^* mutant larval epidermis and CNS, adult brain (2 d old), DA neurons at 2 and 30 d old, and adult flight muscle (2 d old), as indicated. Mitolysosomes are evident as GFP-negative/mCherry-positive (red-only) puncta. Bars, 10 µm. Charts show quantification of mitolysosomes and show mean ± SD of *n* = 6 animals. Number of cells analyzed: epidermis, WT = 29 cells, *Pink1^B9^* = 29 cells; larval CNS, WT = 28 cells, *Pink1^B9^* = 26 cells; adult brain, WT = 74 cells, *Pink1^B9^* = 66 cells; DA neurons 2 d, WT = 100 cells, *Pink1^B9^* = 54 cells and 30 d, WT = 78 cells, *Pink1^B9^* = 82 cells. Quantification of WT control samples is the same as shown in Figs. 1 and 5. Statistical significance determined by Welch’s *t* test; ns, nonsignificant. Genotypes analyzed were *Pink^B9^/Y;da-GAL4/UAS-mito-QC* (epidermis and larval CNS), *Pink^B9^/Y;UAS-mito-QC/+;nSyb-GAL4/*+ (adult brain), *Pink^B9^/Y;UAS-mito-QC/+;TH-GAL4/*+ (DA neurons), and *Pink^B9^/Y;Mef2-GAL4/UAS-mito-QC* (adult muscle).

**Figure 5.**
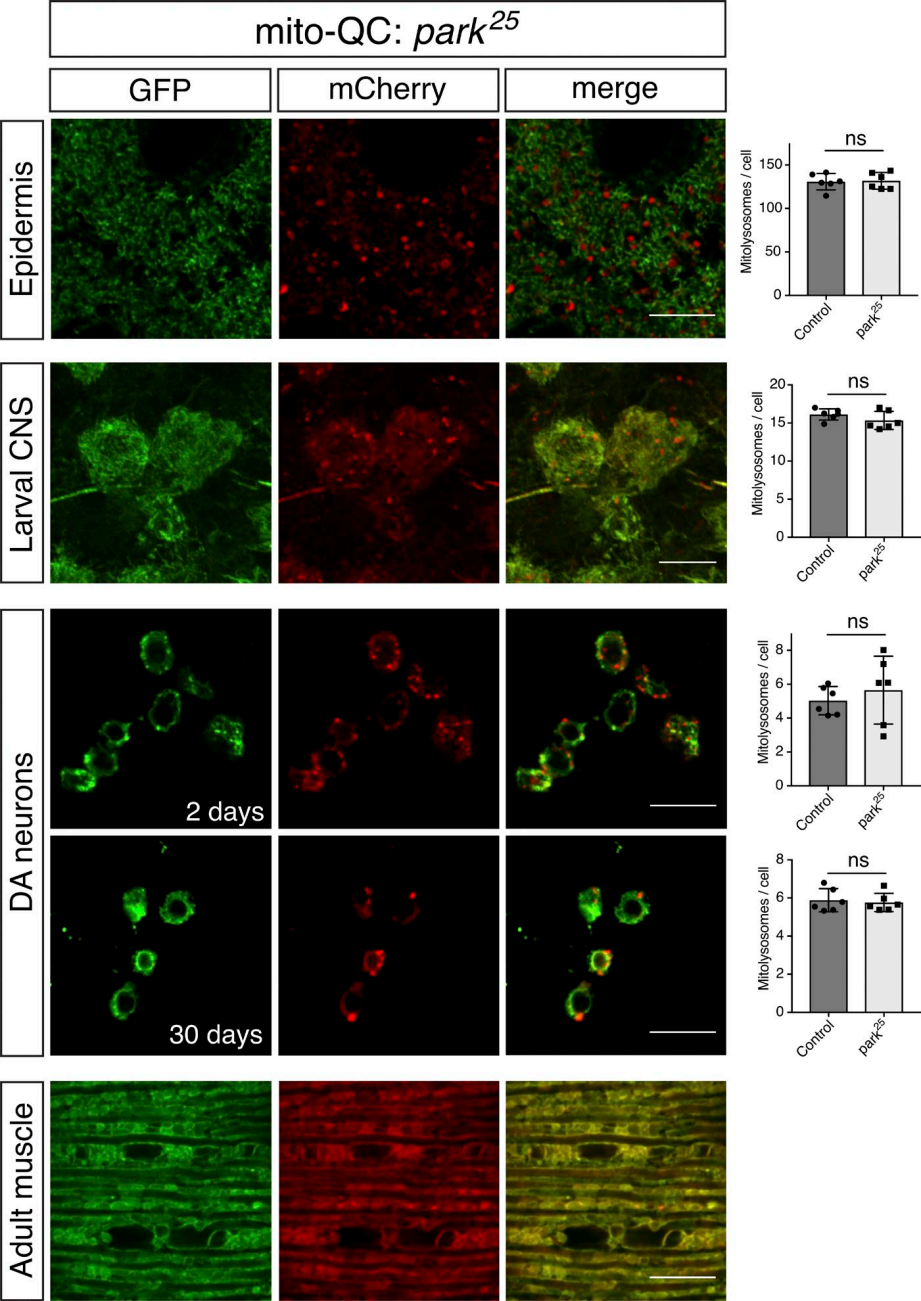
**Basal mitophagy is minimally affected in *parkin* mutants.** Confocal microscopy analysis of mito-QC reporter in *park^25^* larval epidermis and CNS, DA neurons at 2 and 30 d old, and adult flight muscle (2 d old), as indicated. Mitolysosomes are evident as GFP-negative/mCherry-positive (red-only) puncta. Bars, 10 µm. Charts show quantification of mitolysosomes and show mean ± SD of *n* = 6 animals. Quantification of WT control samples is the same as shown in Figs. 1 and 4. Number of cells analyzed: epidermis, WT = 29 cells, *park^25^* = 30 cells; larval CNS, WT = 28 cells, *park^25^* = 31 cells; DA neurons 2 d, WT = 100 cells, *park^25^* = 69 cells and 30 d, WT = 78 cells, *park^25^* = 83 cells. Statistical significance determined by Welch’s *t* test; ns = nonsignificant. Genotypes analyzed were *park^25^*,*da-GAL4/park^25^,UAS-mito-QC* (epidermis and larval CNS), *UAS-mito-QC; park^25^*,*TH-GAL4/park^25^* (DA neurons), and *park^25^*,*Mef2-GAL4/park^25^,UAS-mito-QC* (adult muscle).

Mammalian PINK1 and parkin have been described to play important roles in toxin-induced mitophagy; however, the impact of PINK1 and parkin on basal mitophagy in vivo has not been extensively studied. To address this, we combined our mitophagy reporters with well-characterized *Pink1* and *parkin Drosophila* mutants. Analyzing the previously described tissues where mitolysosomes were abundant, we found that mitophagy was not dramatically reduced in genetic null mutants for *Pink1* (Fig. 4) or *parkin* (Fig. 5). To quantify the degree of mitophagy we applied a semiautomated quantification analysis to segment 3D images to identify and quantify bona fide mitolysosomes (Fig. S2). Quantitative analyses revealed there was no statistical difference in number of mitolysosomes between WT and *Pink1* or *parkin* mutant animals for most cell types, except for larval CNS, where the difference in *Pink1* mutants just reached significance (Figs. 4 and 5). To further verify this unexpected lack of effect, we qualitatively assessed mt-Keima in *Pink1* mutants, and we also observed no gross difference in mitophagy signal (Fig. S3).

Finally, although mitolysosomes were not detectable in WT flight muscle, we considered that because profound mitochondrial disruption occurs in flight muscle of *Pink1/parkin* mutants, mitophagy may be induced as a homeostatic response. However, we again observed no mitophagy signal in *Pink1* or *parkin* mutants (Figs. 4 and 5). Thus, collectively these data indicate that loss of Pink1 or parkin does not substantially influence basal mitophagy in neuromuscular tissues.

The notion that PINK1 and parkin cooperate to mediate bulk degradation of mitochondria has become a dominant concept in the field of PD pathogenesis. Although a large body of work has elucidated the molecular details of PINK1/parkin-mediated stress-induced mitophagy in vitro, it has been an open question whether PINK1 and parkin perform equivalent functions under physiological conditions (Cummins and Gotz, 2017; Rodger et al., 2017; Whitworth and Pallanck, 2017). We have made use of two previously devised mitophagy reporter constructs to visualize mitophagy in fly models for the first time. Our observations reveal that basal mitophagy is highly prevalent in multiple *Drosophila* tissues; however, this mitophagic process is essentially unaffected by null mutations in *Pink1* or *parkin*. Importantly, while this work was under review, a complementary study was published reporting the effect on the mito-QC reporter system in *Pink1* knockout mice (McWilliams et al., 2018). This study reached essentially the same conclusion as our study that basal mitophagy is unaffected by loss of PINK1 in most tissues. Together, our complementary studies analyzing independent mitophagy reporters in multiple mutant models provide strong evidence that the minimal impact of PINK1 and parkin on basal mitophagy is likely a conserved phenomenon.

The simplest interpretation of these results is that PINK1 and parkin do not in fact play an important role in basal mitophagy; however, this seems unlikely given the plethora of evidence supporting PINK1/parkin in some form of mitochondrial degradation. Several alternative explanations can be considered. One clear possibility is that another mode of mitophagy can be induced as a compensatory mechanism. Indeed, there is some precedence for this as it was recently reported that MUL1 acts redundantly alongside parkin (Yun et al., 2014; Rojansky et al., 2016). Other ubiquitin ligases have also been implicated in mitophagy, such as ARIH1/HHARI (Villa et al., 2017). Moreover, although germline knockout of murine *PINK1* and *parkin* has been shown to have very mild phenotypes (Lee et al., 2012), conditional postnatal loss of PINK1 or parkin has revealed robust loss of DA neurons (Shin et al., 2011; Lee et al., 2017). The authors propose that in germline knockouts an unknown mechanism is induced during development that compensated for loss of PINK1/parkin. One possibility that would fit this scenario is induction of an alternative mitophagy pathway or up-regulation of other mitochondrial quality-control processes. Clearly, more needs to be learned about the complex regulatory mechanisms governing mitochondrial turnover under physiological conditions, and our *Drosophila* model offers an excellent system to genetically address these mechanisms.

Another obvious interpretation of the current findings is that basal mitophagy may reflect a distinct homeostatic mechanism from that in which PINK1/parkin function. Indeed, whether the observed basal mitophagy is operating as a quality control mechanism or some other homeostatic process, such as maintenance of mitochondrial levels, is unknown. As previously described, the majority of studies analyzing PINK1/parkin mitophagy have used exogenous stressors and parkin overexpression, leaving it unclear what, if any, physiological stimuli provoke the same PINK1/parkin response. Supporting the requirement for a “second hit,” DA neurons become selectively vulnerable to loss of parkin when mitochondrial DNA mutations are elevated (Pickrell et al., 2015). We have not yet systematically assessed the kinetics of these mitophagy reporters, but we note that the observed mitolysosomes have not been detectably dynamic within the range 10–15 min of live imaging. Our primary motivation was to analyze physiological, basal mitophagy but it may be informative to assess the feasibility of monitoring stress- or toxin-induced mitophagy in vivo. It is possible that loss of parkin or Pink1 may slow the kinetics of this accelerated process but the timescales involved (10–24 h in cell culture) may still be too challenging for in vivo studies. A more targeted stimulus such as light induced reactive oxygen species generation may be more amenable (Yang and Yang, 2013; Ashrafi et al., 2014).

Growing evidence indicates that PINK1/parkin-mediated turnover of mitochondrial components may occur in a piecemeal fashion. For instance, multiple studies have described the selective targeting of portions of mitochondria for turnover (Hämäläinen et al., 2013; Yang and Yang, 2013; McLelland et al., 2014, 2016; Burman et al., 2017), a process likely occurring via so-called mitochondria-derived vesicles (Sugiura et al., 2014). That selective degradation of mitochondrial components may also occur in vivo is supported by compelling evidence using a mass spectrometric method to analyze isotope labeled mitochondrial protein turnover in flies (Vincow et al., 2013). It is possible that the current microscopy approach for the reporters described here is not sufficient to detect mitochondrial component turnover on this scale and so we may miss this aspect of *Pink1/parkin-*dependent quality control. In light of this, it is clear that further work is needed to define the conditions in which PINK1 and parkin promote mitochondrial homeostasis. Notably, it will be important to determine the nature of physiological signals triggering PINK1/parkin mitochondrial turnover. The mitophagy reporter lines described here provide new model systems for studying physiological mitophagy and can be potentially used for screening for novel regulators. Greater insights into the physiological function of PINK1/parkin-mediated mitochondrial homeostasis will allow a clearer understanding of the pathogenic causes of PD and thus a better prospect for more rational therapeutic interventions.

## Materials and methods

### *Drosophila* stocks, husbandry, and locomotor assay

Flies were raised under standard conditions at 25°C on food consisting of agar, cornmeal, molasses, propionic acid, and yeast in a 12-h:12-h light/dark cycle. *Pink1^B9^* mutants were a gift from J. Chung (Seoul National University, Seoul, South Korea; Park et al., 2006), and the *park^25^* mutants have been described previously (Greene et al., 2003). The following strains were obtained from the Bloomington *Drosophila* Stock Center (RRID:SCR_006457): *da-GAL4* (RRID:BDSC_55850), *nSyb-GAL4* (RRID:BDSC_68222), *Mef2-GAL4* (RRID:BDSC_27390), *Atg5*-RNAi (RRID:BDSC_34899), and *TH-GAL4* (RRID:BDSC_8848). In locomotor assays, climbing (negative geotaxis assay) and flight ability was assessed as previously described, with minor modifications (Greene et al., 2003). Transgenic expression was induced by *da-GAL4*, and adult male flies were tested 1–3 d after eclosion. Because X chromosome nondisjunction is present in multiple balanced *Pink1^B9^* mutant stocks, correct genotypes were determined by either combining paternal animals with X chromosome markers or by PCR-based genotyping of discarded tissue after dissection. For deferiprone treatment, mito-QC– or mt-Keima–expressing animals were raised on normal food dosed with deferiprone (catalog number D1720; LKT Laboratories) to a final concentration of 65 µM.

### Transgenic line construction

A plasmid containing the mito-QC sequence was obtained from I. Ganley (University of Dundee, Dundee, Scotland, UK) through the Medical Research Council Protein Phosphorylation and Ubiquitylation Unit Reagents and Services facility (College of Life Sciences, University of Dundee, Scotland; pBabe.hygro-mCherry-GFP FIS 101–152(end) [DU40799]; Allen et al., 2013). PCR primers containing EcoRI and XbaI restriction sites were used to amplify mito-QC, and after restriction digestion and purification by standard methods, mito-QC was cloned into complementary sites in a pUAST.attB transgenesis vector. Constructs were verified by sequencing before sending for transgenesis by BestGene. UAS-mito-QC was integrated into attP16 and attP2 sites and verified by PCR. Several transformant lines were tested for consistency before selecting a single line of each integration site for further study.

mt-Keima (Katayama et al., 2011) was amplified and cloned from mt-Keima (h)-pIND(SP1) plasmid into *Drosophila* expression vector pUASt. pUASt-mt-Keima construct was injected and recombined in *w^1118^* fly embryos at BestGene. Four independent UAS-mt-Keima strains were selected: UAS-mt-Keima M7 (II), UAS-mt-Keima M3 (II), UAS-mt-Keima M2 (III), and UAS-mt-Keima M4 (III) based on detection of fluorescent mt-Keima signal in larval eyes by crossing them with GMR-GAL4. UAS-mt-Keima M2 flies were genetically recombined with Tub-GAL4/TM3 (III) flies to finally generate Tub-GAL4, UAS-mt-Keima M2/TM3.

### Antibodies and dyes

The following antibodies and dyes were used: ATP5A (catalog number ab14748, RRID:AB_301447; 1:500; Abcam), Actin (catalog number MAB1501, RRID:AB_2223041; 1:5,000; Millipore), GFP (catalog number ab290, RRID:AB_303395; 1:2,000; Abcam), DsRed (catalog number 632496, RRID:AB_10013483; 1:1,000; Clontech Laboratories), Keima-Red (catalog number M126-3, RRID:AB_10210643; 1:1,000; MBL International), α-Tubulin (catalog number T6793, RRID:AB_477585; 1:5,000; Sigma-Aldrich), and 500 nM LysoTracker Deep Red (L12492; Invitrogen). The following secondary antibodies were used for immunoblotting: anti-rabbit HRP (catalog number G-21234, RRID:AB_2536530; 1:10,000; Thermo Fisher Scientific), anti-mouse HRP (catalog number ab67891, RRID:AB_1140250; 1:10,000; Abcam). The following secondary antibody were used for immunofluorescence: anti-mouse AF647 (catalog number A-21235, RRID:AB_2535804; 1:200; Thermo Fisher Scientific).

### Immunoblotting

Protein samples isolated from whole adult fly were prepared in lysis buffer (50 mM Tris·HCl, 150 mM NaCl, 10% [vol/vol] glycerol, 1% Triton X-100, 10 mM N-ethylmaleimide, 2 mM EGTA, 1 mM, MgCl_2_, 50 µM MG-132, and protease inhibitor mixture; Roche), resolved by SDS-PAGE using 12% precast gels (Bio-Rad), and transferred onto nitrocellulose membrane (Bio-Rad) using a Bio-Rad Transblot Transfer Apparatus. Membranes were blocked with 5% skimmed milk in TBS with 0.1% Tween-20 for 1 h at RT and probed with the appropriate primary antibodies diluted in the blocking solution overnight at 4°C. Membranes were washed repeatedly in TBS with 0.1% Tween-20, and then the appropriate HRP-conjugated secondary antibodies (Dako) were incubated for 1 h at RT. Detection was achieved with ECL-Prime detection kit (Amersham). As a loading control, membranes were incubated with anti–α-Tubulin or anti–Actin antibodies for 1 h at RT.

### Immunohistochemistry and sample preparation

For immunostaining of mito-QC and mt-Keima, larval epidermis or adult flight muscle were dissected in PBS and fixed in 4% formaldehyde, pH 7.0, for 30 min, permeabilized in 0.3% Triton X-100 for 30 min, and blocked with 0.3% Triton X-100 plus 1% bovine serum albumin in PBS for 1 h at RT. Tissues were incubated with ATP5A antibody diluted in 0.3% Triton X-100 plus 1% bovine serum albumin in PBS overnight at 4°C, rinsed three times 10 min with 0.3% Triton X-100 in PBS, and incubated with the appropriate fluorescent secondary antibodies for 2 h at RT. The tissues were washed twice in PBS and mounted on slides using Prolong Diamond Antifade mounting medium (Thermo Fischer Scientific).

For mitolysosome analysis of mito-QC, tissues were dissected in PBS and fixed in 4% formaldehyde, pH 7.0, for 30 min, rinsed in PBS, mounted in Prolong Diamond Antifade mounting medium (Thermo Fischer Scientific), and generally imaged the next day. For mitolysosome analysis of mt-Keima, larvae were dissected and mounted in Prolong Diamond Antifade mounting medium (Thermo Fischer Scientific) for immediate live imaging. Tissues were imaged via sequential excitations (458 nm, green; 561 nm, red) being captured at 578- to 638-nm emission range.

### LysoTracker assay

Larvae were dissected in PBS. The epidermal cells were stained for 2 min with LysoTracker Deep Red diluted in PBS (final concentration of 500 nM). Samples were mounted in PBS immediately visualized by confocal microscopy (live imaging).

### Microscopy

Fluorescence imaging was conducted using a Zeiss LSM 880 confocal microscope equipped with Nikon Plan-Apochromat 40×/1.3 NA and 63×/1.4 NA oil immersion objectives or an Andor Dragonfly spinning disk confocal microscope equipped with a Nikon Plan-Apochromat 100×/1.45 NA oil immersion objective. Z-stacks were acquired at 0.5-µm steps.

### Image analysis and quantification

Confocal images were processed using Fiji (ImageJ) software (RRID:SCR_002285) for figure presentation. For quantification of mitolysosomes, spinning disk microscopy–generated images were processed using Imaris (version 9.0.2) analysis software (BitPlane; RRID:SCR_007370) to identify and count individual red-only puncta. The GFP and mCherry signals were adjusted to reduce background noise and retain only the distinct mitochondria network and red puncta, respectively. A surface rendered 3D structure corresponding to the mitochondria network was generated using the GFP signal. This volume was subtracted from the red channel to retain the mCherry signal that did not colocalize with the GFP-labeled mitochondria network. The mitolysosome puncta were selected according to their intensity and an estimated size of 0.5 µm diameter, previously measured with Imaris. Additionally, the puncta were filtered with a minimum size cutoff of 0.2 µm diameter. The remaining puncta were counted as the number of mitolysosomes.

Epidermal cells and larval CNS soma were analyzed individually where discrete cells could be distinguished. For DA neurons and adult CNS soma, small groups of cells were analyzed together and a mean score calculated per cell.

The mean number of mitolysosomes per cell was calculated per animal. Data points in the quantification charts show mean mitolysosomes per cell for individual animals, where *n* = 6 animals for each condition.

### Statistical analysis

For behavioral analyses, a Kruskal–Wallis nonparametric test with Dunn’s post-hoc correction for multiple comparisons was used. The number of mitolysosomes was analyzed by Welch’s *t* test. Analyses were performed using GraphPad Prism 7 software (RRID:SCR_002798).

### Online supplemental material

Fig. S1 shows that expression of mito-QC and mt-Keima is benign in *Drosophila*. Fig. S2 shows mitolysosome quantification workflow for larval epidermis (mito-QC only). Fig. S3 shows mitolysosome analysis with mt-Keima in Pink1 mutant larval tissues.

## Supplementary Material

Supplemental Materials (PDF)
