# Supplementary material for: Basal mitophagy is widespread in Drosophila but minimally affected by loss of Pink1 or parkin
Source: J Cell Biol. 2018 May 7;217(5):1613–22. doi: 10.1083/jcb.201801044 (PMC5940313; doi:10.1083/jcb.201801044)
Supplement: Supplemental Materials (PDF) [file JCB_201801044_sm.pdf]

Supplemental material

Lee et al., https://doi.org/10.1083/jcb.201801044

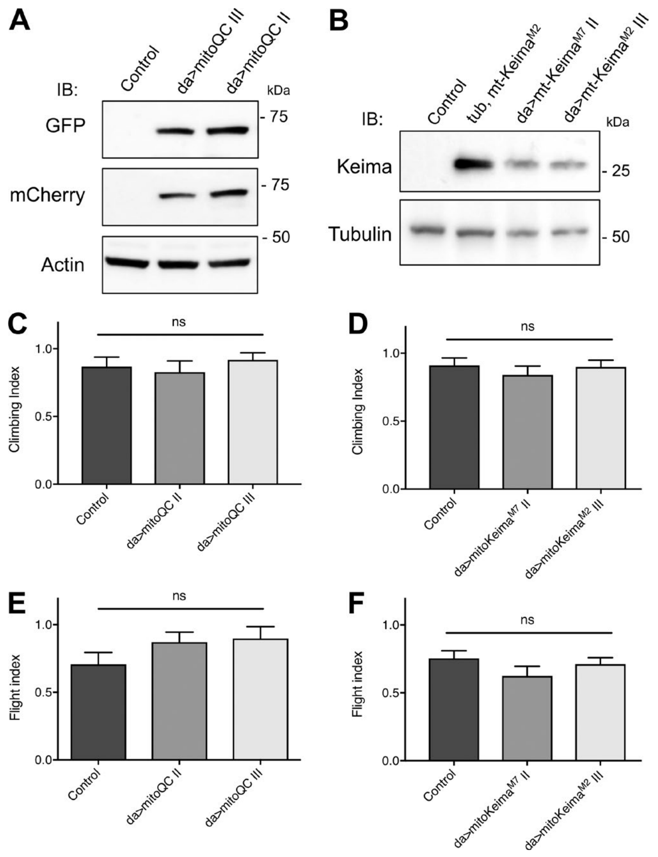

Figure S1. **Expression of mito-QC and mt-Keima is benign in *Drosophila*. (A and B)** Expression analysis of independent transgenic lines expressing (A) mito-QC and (B) mt-Keima induced by tubulin (tub)- or daughterless (da)-GAL4 lines. Immunoblots were probed with the indicated antibodies. Control genotype is nontransgenic w^{1118}. **(C–F)** Locomotor assays for climbing (C and D) and flight ability (E and F) of the same genotypes analyzed above. Charts show mean ± 95% confidence interval. *n* > 50 animals (2 d old). Statistical analysis was determined by Kruskal–Wallis nonparametric test with Dunn's post-hoc correction for multiple comparisons; ns, nonsignificant. Control genotype for behavior is *da-GAL4/+*.

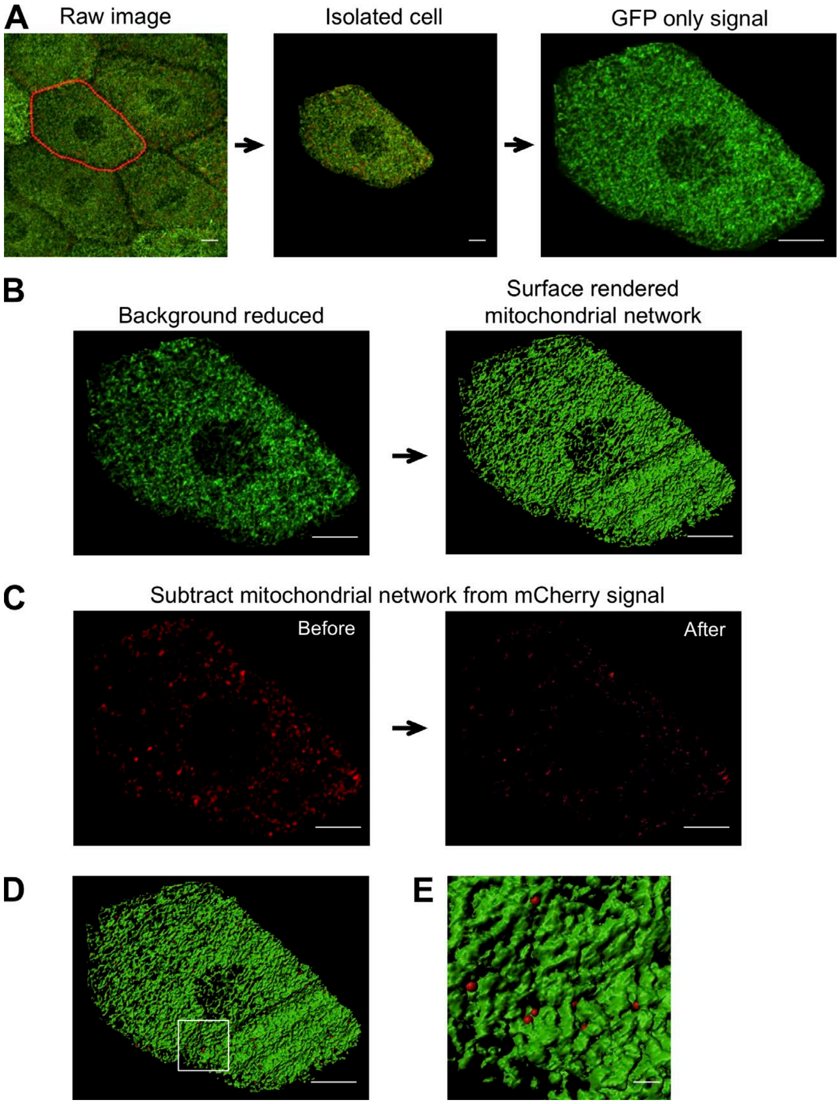

Figure S2. **Mitolysosome quantification workflow for larval epidermis (mito-QC only).** Z-stack images were acquired by spinning disk microscopy for quantification. **(A)** Where possible, individual cells were isolated for workflow analysis. **(B)** The contrast of the mitochondrial network (GFP only) was adjusted to reduce the background, and a rendered 3D surface corresponding to the mitochondrial network was generated. **(C)** This surface was then subtracted from the mCherry channel to discard the mCherry signal overlapping the GFP-labeled mitochondrial network, leaving the red-only puncta. **(D and E)** Imaris-defined mitolysosomes (red) outside of the mitochondrial network (green). Genotype shown is *da-GAL4/UAS-mito-QC*. Bars: (A–D) 10 µm; (E) 2 µm.

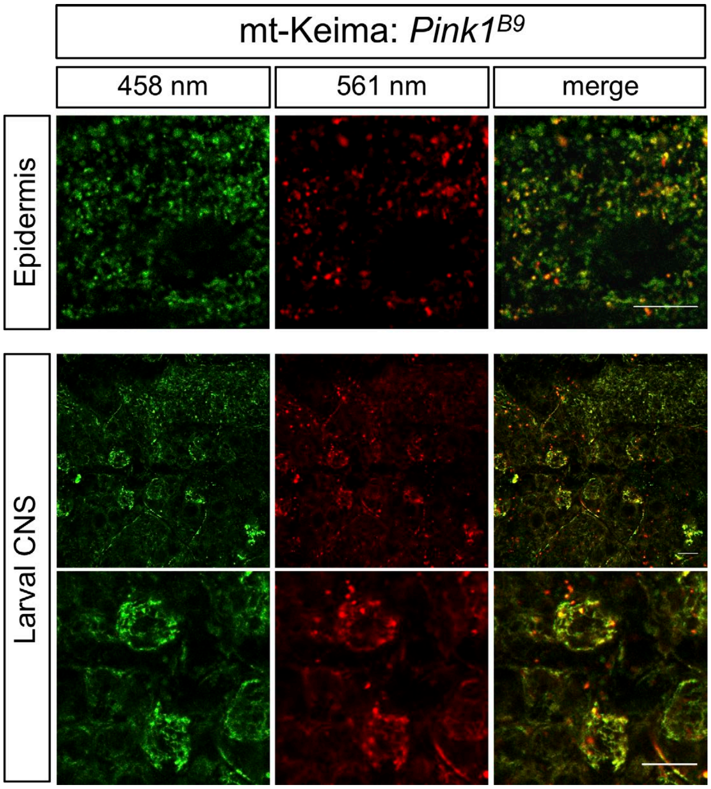

Figure S3. **Mitolysosome analysis with mt-Keima in *Pink1* mutant larval tissues.** Live confocal microscopy of larval epidermal cells and ventral ganglion of the CNS to visualize mt-Keima in *Pink1*^{B9} mutant animals (compare with Fig. 2). Genotype analyzed is *Pink1*^{B9}*; tub-GAL4, UAS-mt-Keima.* Bars, 10 µm.
